# Diagnosis and Assessment of Apathy in Elderly Chinese Patients With Cerebral Small Vessel Disease

**DOI:** 10.3389/fpsyt.2021.688685

**Published:** 2021-08-03

**Authors:** Hóngyi Zhào, Yu Liu, Zhenxi Xia, Hongyang Xie, Yonghua Huang

**Affiliations:** ^1^Department of Neurology, Seventh Medical Center of Chinese PLA General Hospital, Beijing, China; ^2^Department of Psychiatry, NO 984 Hospital of People's Liberation Army, Beijing, China; ^3^Department of Neurology, NO 984 Hospital of People's Liberation Army, Beijing, China

**Keywords:** cerebral small vessel disease, apathy, neuropsychiatric disorder, aging, depression

## Abstract

**Objective:** The study aimed to estimate the frequency of apathy in Chinese patients with cerebral small vessel disease (CSVD) and investigate the relationship between apathy and neuroimaging markers of CSVD.

**Methods:** A total of 150 CSVD aged patients were recruited for a cross-sectional observational study. Following the new revised version of diagnostic criteria for apathy (DCA), each patient was evaluated successively by the neuropsychiatric inventory (NPI-apathy), geriatric depression scale (GDS), and caregiver burden scale (CBS). The MRI presence of lacunes, white matter hyperintensities, cerebral microbleeds, and perivascular spaces were rated independently. Furthermore, presence of all these MRI markers were summed in a score of 0–4 representing all CSVD features combined.

**Results:** According to the DCA, we found that the frequency of apathy in Chinese Alzheimer's disease patients reached 37.33%, with lack of and diminished goal-directed activities in the dimension of behavior/cognition. We did not find a close relationship between apathy and depression. Caregiver burden was positively correlated with apathy severity. Apathy, but not depression, was positively associated with total CSVD burden, rather than a separate MRI marker of CSVD.

**Conclusion:** As a key component of neuropsychiatric symptoms, apathy was common in Chinese elderly with CSVD, more attention should be paid to apathy in clinical practice of CSVD.

## Introduction

Cerebral small vessel disease (CSVD) refers to a group of pathological processes which affect the small arteries, arterioles, venules, and capillaries of the brain (1). The features of CSVD range from asymptomatic radiological markers occurrence to symptomatic characteristics including cognitive deficits and gait decline (2). Recent studies reported that neuropsychiatric symptoms (NPS) are quite common in CSVD patients (3–5).

In major contributions, apathy was defined as a lack of motivation that persists over time and causes identifiable functional impairment (6). It has been confirmed that apathy can be found in many neurological diseases, such as Alzheimer's disease (AD), Parkinson disease (PD), Huntington disease (HD), etc., (7–9). Based on the vast sub-cortical changes in CSVD patients that overlap with undermined brain circuits in apathy (10), vascular apathy was hypothesized recently (11). Tay et al. (12) proposed that apathy found in CSVD patients might be a disconnection syndrome, which was driven by disruption of white matter tracts connecting regions important for motivation. In detail, the connections between frontal-subcortical circuits, including the anterior cingulate cortex, might be responsible for the initiation of goal-directed behaviors (13). Furthermore, it is reported that the presence of apathy significantly affects the patient's quality of life (14), and the severity of apathy is associated with a faster cognitive and functional decline in neurodegenerative diseases (15).

However, apathy is under-recognized and has been poorly understood in cerebrovascular disease clinical practice for a long time, although, the occurrence of lack of motivation reached one-third in patients after stroke (16). Apathy was easily neglected, even misconstrued as depression among post-stroke patients (17). In the last decade, along with the considerable advances in the domain of apathy in brain disorders, including the apathy biological- and neural-based, the consensus of diagnosis and assessment of apathy has been revised several times (6, 18). According to the latest version of diagnostic criteria of apathy (DCA) in brain disorders proposed in 2018 (Nice, France), the spectrum of apathy was enlarged into three dimensions: lack of or diminished goal-directed behavior-cognition, emotion, and social interaction. The purpose of the present study was to investigate apathy and the relevant phenotype in elderly Chinese patients with CSVD, and to investigate the relationship between apathy severity and cognitive disorders in aged CSVD patients.

## Methods

### Participants

We conducted a clinical cross-sectional observational study from January 1, 2019 to April 1, 2020 and recruited 150 elderly patients with CSVD consecutively from the Department of Neurology at the Seventh Medical Center of PLA General Hospital (Beijing, China). Our study was approved by the Academic Ethics Committee of the Biological Sciences Division of PLA General Hospital in Beijing, China.

The exclusion criteria were: patients with major stroke or cerebral bleeding episodes; other causes of leukoencephalopathy (e.g., immune, demyelination, genetic); major psychiatric diseases; use of psychotropic medications; multisystem diseases such as polyarteritis nodosa, nervous system vasculitis associated with connective tissue disorders, vasculitis secondary to infectious, etc.; arthritis; MRI contraindications; and neurodegenerative dementia.

### Magnetic Resonance Imaging Measurements

A 3.0T MRI brain (Discovery MR750; GE Healthcare, USA) scan displayed white matter lesions reflecting the degree of SVD. A brain MRI (slice and interslice thicknesses of 5 and 1.5 mm, respectively) was carried out as follows: T1 fluid-attenuated inversion recovery (TR, 1750 ms; TE, 23 ms; T1, 780 ms; FOV, 24 cm) and T2-weighted imaging (TR, 7498 ms; TE, 105 ms; FOV, 24 cm) sequences. The assessors were blinded to imaging findings.

### Total CSVD Burden Score

The total CSVD burden score was calculated according to Chen et al. (19). Briefly, one point was allocated to each of the following MRI parameters: severe WMH (periventricular WMH Fazekas 3 or deep WMH Fazekas 2–3), presence of lacunes, microbleeds, and moderate to severe BG-PVS (semi-quantitative rating > 1), with total scores ranging from 0 to 4 points.

### Diagnosis and Assessment of Apathy

Each patient was diagnosed according to the DCA (6) (shown in Supplementary Material 1), and assessed by NPI-apathy, geriatric depression scale (GDS), mini-mental evaluation scale (MMSE), and care-giver burden scale (CBS). Information was collected from caregivers if necessary. All assessments were conducted according to the Chinese version of the above guidelines, and data were organized and completed in the following 24 h.

### Statistical Analysis

Differences between the groups' clinical and demographic data were analyzed by using one-way analysis of variance. Bivariate correlation was selected to detect the correlation between CBS and neuropsychiatric disorders, as well as the relationship between severity of apathy and depression. Stepwise multiple linear regression was used to investigate the correlation between apathy/depression severity and CSVD biomarkers, controlling for age, sex, education, and MMSE score. The significance threshold was set at *P* < 0.05 in all statistical tests. Analysis was carried out using SPSS 22.0 software.

## Results

According to the DCA, the frequency of apathy in Chinese AD patients was 37.33%. Table 1 demonstrates the demographic characteristics of our subjects. The apathetic group scored 21.05 ± 4.09 in MMSE, compared to 24.58 ± 4.06 in the non-apathetic group. There were significant differences in the scores between apathetic and non-apathetic groups (*P* = 0.000). Meanwhile, apathetic group patients showed more obvious CSVD burden severity relative to non-apathetic group individuals (CSVD burden score: 2.09 ± 1.00 vs. 1.65 ± 0.84, *P* = 0.004).

**Table 1:** Clinical and demographic characteristics of the subjects with and without apathy.

	**Overall (*N* = 150)**	**Apathy+ (*N* = 56)**	**Apathy– (*N* = 94)**	***P*-value**
Men, %	79 (52.66%)	27 (48.21%)	52 (55.32%)	0.399
Age, years	70.59 (8.02)	72.00 (8.57)	65.76 (7.59)	0.097
Education, years	8.49 (3.47)	8.11 (3.71)	8.72 (3.32)	0.294
NPI-apathy	3.86 (4.05)	6.68 (3.45)	2.18 (3.40)	0.000^#^
MMSE score	23.43 (4.32)	21.05 (4.09)	24.58 (4.06)	0.000^#^
CSVD burden score	1.81 (0.92)	2.09 (1.00)	1.65 (0.84)	0.004^#^
Fazekas score	1.95 (0.91)	2.13 (0.83)	1.84 (0.94)	0.056

*Mean (standard deviation). MMSE, Mini-mental state evaluation; CSVD, Cerebral small vessel disease*.

# *P < 0.05 apathy+ relative to apathy–*.

Furthermore, according to criterion B of DCA, most Chinese apathetic elderly CSVD patients exhibited a lack of or diminished goal-directed activities in dimension behavior/cognition (78.57%), followed by goal-directed social interaction (64.28%), and goal-directed emotion (64.28%). Details are listed in Table 2.

**Table 2:** Presence of CSVD aged patients in each apathetic dimension according to DCA.

**Dimension**	***N***	**B1-behavior/cognition *N* (%)**	**B2-emotion *N* (%)**	**B3-social interaction *N* (%)**
Apathy+	56	44 (78.57%)	29 (51.78%)	36 (64.28%)
Apathy–	94	10 (10.64%)	7 (7.45%)	5(5.32%)

The severity of depression was evaluated by GDS, and distance correlation of GDS score and NPI-apathy was only 0.158 (*P* = 0.054) which indicated a low correlation between apathy and depression.

Caregiver burden was indicated by CBS. The correlation coefficient between CBS score and NPI-apathy, MMSE, and GDS score was 0.485, −0.319, and 0.300, respectively. This result indicated that caregiver burden was most closely correlated with apathy severity in aged CSVD patients.

Lastly, stepwise multiple linear regression was adopted to analyze the relationship between apathy severity and MRI markers of CSVD such as WMH, LI number, CMB number, BG-PVS score, and total CSVD burden score, controlling for age, sex, education, and MMSE score. We only found that NPI-apathy score was closely associated with total CSVD burden (R^2^ = 0.332, t = 4.134, *P* < 0.000). Whereas, GDS score was not significantly associated with total CSVD burden (R^2^ = 0.036, t = 0.364, *P* = 0.674) and other specific CSVD MRI markers.

## Discussion

Apathy has gained more and more attention in recent years as one of the most common neuropsychiatric symptoms associated with CSVD (20), although, the prevalence of apathy has not reached a consensus. The prevalence of apathy in the general population (≥50 years) was estimated at 23.7% (21). Diagnosed based on neuropsychiatric inventory assessment, Reyes et al. (22) found that apathy frequency was 41% in cerebral autosomal dominant arteriopathy with subcortical infarcts and leukoencephalopathy (CADASIL) patients (a genetic model of CSVD). In the current study, we revealed that the prevalence of apathy was 37.33% in Chinese aged patients with sporadic CSVD. These distinct findings of apathy frequency might be caused by the choices of diagnostic methods. Owing to the revision of DCA, more consistency studies of apathy in neuropsychiatric disorders can emerge in future. As has been mentioned by Robert et al. (6), goal-directed activities classified in criterion B of DCA were changed from domains of “behavior, cognition, and emotion” to dimensions of “behavior/cognition, emotion, and social interaction.” According to the renewed criterion B of DCA, most Chinese apathetic elderly CSVD patients exhibited loss of or diminished goal-directed activities in dimension behavior/cognition, followed by goal-directed social interaction, and goal-directed emotion.

Depression is another major neuropsychiatric symptom in cerebrovascular disease (3). As far as we know, there is plenty of literature that has found a relationship between depression and CSVD. Vascular depression was proposed by Alexopoulos (23) even earlier than vascular apathy. CSVD patients showed depressive symptoms if the subcortical limbic structure was involved (24). Also, vascular depression was reported to be associated with “depression-executive dysfunction syndrome” (25). However, apathy and depression are dissociable, in spite of the existence of several overlapping features, such as loss of pleasure and reduced energy (16). Apathy and depression should be differentiated in clinical practice, since apathy, but not depression, was associated with executive dysfunction in CSVD (26). Similar findings were also detected in AD patients in our previous study (7). In our current study, we only found an average GDS score of 6.56 ± 4.60, which implied that depressive symptoms were not severe in aged CSVD patients. Meanwhile, GDS score was not significantly associated with total CSVD burden or other specific CSVD MRI markers. These findings were not similar to the conclusions of Pasi et al. (27) and Direk et al. (28), which supported a vascular depression hypothesis in CSVD. The main explanation might be that the subjects we recruited were inpatients with more severe cognitive deficits (MMSE: 23.43 ± 4.32). As has been mentioned by Mortby et al. (24), “apathy become[s] universal among the severely cognitively impaired, depression is an initial symptom, already apparent in the pre-clinical stage of MCI.”

Actually, investigators have confirmed that the underlying mechanisms of apathy and depression in CSVD are distinct. For example, Eurelings et al. (29) concluded that increased C-reactive protein levels (a low-grade inflammation) were associated with apathy symptoms but not with depressive symptoms. At the same time, Hollocks et al. (13) managed to revealed that white matter microstructural changes in small vessel disease are associated with apathy but not directly with depressive symptoms.

In the present study, we did not detect a significant association between apathy severity and MRI markers of CSVD, except CSVD burden score. The definition of “total CSVD score” was developed by the Maastricht collaborative group to estimate CSVD burden by summing up the presence of lacunes, white matter hyperintensities, cerebral microbleeds, and perivascular spaces independently into an ordinal score (30). As we know, these MRI markers of CSVD do not occur separately, and Wardlaw et al. (31) suggested searching for methods to assess the total CSVD load on imaging in order to avoid over-reliance on one feature only. This might explain why we did not find a statistically close relationship between apathy severity and separate MRI markers of CSVD.

We also found that apathetic group individuals exhibited a more obvious cognitive decline in comparison with the non-apathetic group. In addition, caregiver burden of apathetic group patients was markedly higher than non-apathetic group subjects. These results were consistent with many other reports that have demonstrated that the severity of apathy correlates with caregiver burden in patients with AD, frontotemporal dementia, amyotrophic lateral sclerosis, etc., (7, 32–34). The increase of caregiver burden might result from a decrease in physical activities and daily living ability popularly found in apathetic patients (32). These findings implied that recognizing, diagnosing, and assessing apathy properly is helpful to alleviate apathy-associated caregiver burden.

Several limitations of this study should be considered. First, the sample size was small. Second, there is emerging evidence that new information and communication technology approaches are capable of providing clinicians with valuable additional information in terms of assessment, and therefore, more accurate diagnosis of apathy including actigraphy devices is possible (35). Whereas, we did not select an objective method to assess apathy in our investigation. In a future study, we will collect more information around apathy.

In conclusion, the prevalence of apathy was quite high in elderly Chinese patients with CSVD. Based on MRI imaging, apathetic patients had a more severe SVD burden. Severity of apathy, rather than depression, was positively associated with CSVD burden. In CSVD aged patients, caregiver burden was closely correlated with apathy severity.

## Data Availability Statement

The original contributions presented in the study are included in the article/Supplementary Material, further inquiries can be directed to the corresponding author/s.

## Ethics Statement

Our study was approved by the Academic Ethic Committee of the Biological Sciences Division of Seventh Medical Center of Chinese PLA General Hospital in Beijing, China. The patients/participants provided their written informed consent to participate in this study.

## Author Contributions

HZ and ZX were responsible for data collection. YL was responsible for manuscript writing. HX was responsible for data analysis. YH was responsible for the study design. All authors contributed to the article and approved the submitted version.

## Conflict of Interest

The authors declare that the research was conducted in the absence of any commercial or financial relationships that could be construed as a potential conflict of interest.

## Publisher's Note

All claims expressed in this article are solely those of the authors and do not necessarily represent those of their affiliated organizations, or those of the publisher, the editors and the reviewers. Any product that may be evaluated in this article, or claim that may be made by its manufacturer, is not guaranteed or endorsed by the publisher.
