# Supplementary material for: Diagnosis and Assessment of Apathy in Elderly Chinese Patients With Cerebral Small Vessel Disease
Source: Front Psychiatry. 2021 Aug 3;12:688685. doi: 10.3389/fpsyt.2021.688685 (PMC8368720; doi:10.3389/fpsyt.2021.688685)
Supplement: Supplementary file 1 [file Data_Sheet_1.docx]

Supplementary Material 1

# Apathy diagnostic criteria 2018.

**CRITERION A:** A quantitative reduction of goal-directed activity either in behavioral, cognitive, emotional or social dimensions in comparison to the patient’s previous level of functioning in these areas. These changes may be reported by the patient himself/herself or by observation of others.

**CRITERION B:** The presence of at least 2 of the 3 following dimensions for a period of at least four weeks and present most of the time

B1. BEHAVIOUR & COGNITION

Loss of, or diminished, goal-directed behaviour or cognitive activity as evidenced by at least one of the following:

**General level of activity:** the patient has a reduced level of activity either at home or work, makes less effort to initiate or accomplish tasks spontaneously, or needs to be prompted to perform them.

**Persistence of activity:** He/she is less persistent in maintaining an activity or conversation, finding solutions to problems or thinking of alternative ways to accomplish them if they become difficult.

**Making choices:** He/she has less interest or takes longer to make choices when different alternatives exist (e.g., selecting TV programs, preparing meals, choosing from a menu, etc.)

**Interest in external issue:** He/she has less interest in or reacts less to news, either good or bad, or has less interest in doing new things

**Personal wellbeing:** He/she is less interested in his/her own health and wellbeing or personal image (general appearance, grooming, clothes, etc.)

B2. EMOTION

Loss of, or diminished, emotion as evidenced by at least one of the following:

**Spontaneous emotions:** the patient shows less spontaneous (self-generated) emotions regarding their own affairs, or appears less interested in events that should matter to him/her or to people that he/she knows well.

**Emotional reactions to environment:** He/she expresses less emotional reaction in response to positive or negative events in his/her environment that affect him/her or people he/she knows well (e.g., when things go well or bad, responding to jokes, or events on a TV program or a movie, or when disturbed or prompted to do things he/she would prefer not to do).

**Impact on others:** He/she is less concerned about the impact of his/her actions or feelings on the people around him/her.

**Empathy:** He/she shows less empathy to the emotions or feelings of others (e.g., becoming happy or sad when someone is happy or sad, or being moved when others need help).

**Verbal or physical expressions:** He/she shows less verbal or physical reactions that reveal his/her emotional states.

B3. SOCIAL INTERACTION

Loss of, or diminished engagement in social interaction as evidenced by at least one of the following:

**Spontaneous social initiative:** the patient takes less initiative in spontaneously proposing social or leisure activities to family or others.

**Environmentally stimulated social interaction:** He/she participates less, or is less comfortable or more indifferent to social or leisure activities suggested by people around him/her.

**Relationship with family members:** He/she shows less interest in family members (e.g., to know what is happening to them, to meet them or make arrangements to contact them).

**Verbal interaction:** He/she is less likely to initiate a conversation, or he/she withdraws soon from it.

**Homebound:** He /She prefer to stays at home more frequently or longer than usual and shows less interest in getting out to meet people.

**CRITERION C:** These symptoms (A - B) cause clinically significant impairment in personal, social, occupational, or other important areas of functioning.

**CRITERION D:** The symptoms (A - B) are not exclusively explained or due to physical disabilities (e.g. blindness and loss of hearing), to motor disabilities, to a diminished level of consciousness, to the direct physiological effects of a substance (e.g. drug of abuse, medication), or to major changes in the patient’s environment.
